# Use of Hypoprothrombinemia-Inducing Cephalosporins and the Risk of Hemorrhagic Events: A Nationwide Nested Case-Control Study

**DOI:** 10.1371/journal.pone.0158407

**Published:** 2016-07-27

**Authors:** Li-Ju Chen, Fei-Yuan Hsiao, Li-Jiuan Shen, Fe-Lin Lin Wu, Woei Tsay, Chien-Ching Hung, Shu-Wen Lin

**Affiliations:** 1 Graduate Institute of Clinical Pharmacy, College of Medicine, National Taiwan University, Taipei, Taiwan; 2 School of Pharmacy, National Taiwan University, Taipei, Taiwan; 3 Departments of Pharmacy, National Taiwan University Hospital, Taipei, Taiwan; 4 Internal Medicine, National Taiwan University Hospital, Taipei, Taiwan; University of Pennsylvania School of Medicine, UNITED STATES

## Abstract

**Objective:**

Existing data regarding the risk of hemorrhagic events associated with exposure to hypoprothrombinemia-inducing cephalosporins are limited by the small sample size. This population-based study aimed to examine the association between exposure to hypoprothrombinemia-inducing cephalosporins and hemorrhagic events using National Health Insurance Research Database in Taiwan.

**Design:**

A nationwide nested case-control study.

**Setting:**

National Health Insurance Research database.

**Participants:**

We conducted a nested case-control study within a cohort of 6191 patients who received hypoprothrombinemia-inducing cephalosporins and other antibiotics for more than 48 hours. Multivariable conditional logistic regressions were used to calculate the adjusted odds ratio (aOR) and 95% confidence interval (CI) for hemorrhagic events associated with exposure to hypoprothrombinemia-inducing cephalosporins (overall, cumulative dose measured as defined daily dose (DDD), and individual cephalosporins).

**Results:**

Within the cohort, we identified 704 patients with hemorrhagic events and 2816 matched controls. Use of hypoprothrombinemia-inducing cephalosporins was associated with increased risk of hemorrhagic events (aOR, 1.71; 95% CI, 1.42–2.06), which increased with higher cumulative doses (<3 DDDs, aOR 1.62; 3–5 DDDs, aOR 1.78; and >5 DDDs, aOR 1.89). The aOR for individual cephalosporin was 2.88 (95% CI, 2.08–4.00), 1.35 (1.09–1.67) and 4.57 (2.63–7.95) for cefmetazole, flomoxef, and cefoperazone, respectively. Other risk factors included use of anticoagulants (aOR 2.08 [95% CI, 1.64–2.63]), liver failure (aOR 1.69 [1.30–2.18]), poor nutritional status (aOR 1.41 [1.15–1.73]), and history of hemorrhagic events (aOR 2.57 [1.94–3.41]) 6 months prior to the index date.

**Conclusions:**

Use of hypoprothrombinemia-inducing cephalosporins increases risk of hemorrhagic events. Close watch for hemorrhagic events is recommended when prescribing these cephalosporins, especially in patients who are at higher risk.

## Key Points

This nested case-control study demonstrated that use of hypoprothrombinemia-inducing cephalosporins was associated with higher risk of hemorrhages in a cohort of 6191 patients. Cefoperazone, cefmetazole, flomoxef, anticoagulants, liver failure, poor nutritional status, and history of hemorrhages increased the risk.

## Introduction

Cephalosporins are commonly prescribed and widely used to prevent or treat various infectious diseases. However, previous studies have suggested a potential correlation between certain cephalosporins and hemorrhagic tendency or events. [1–12] The potential mechanism regarding this risk is that cephalosporins may cause hypoprothrombinemia through inhibition of the growth of vitamin K–producing intestinal bacteria, such as *Bacteroides* spp., *Enterobacteriaceae*, and enterococci [13–15] or inhibition of vitamin K-epoxide reductase and vitamin K-dependent carboxylase. [16–19]

The cephalosporins that have been reported to increase risk of hemorrhagic tendency or events can be categorized into 4 groups based on different side chains attached to 3-position of the cephem nucleus. The first and the largest group of these cephalosporins is N-methylthiotetrazole (NMTT)-side-chain-containing cephalosporins, which includes cefamandole, cefotetan, cefmatazole, moxalactam, and cefoperazone; each of them has been reported to induce hypoprothrombinemia or hemorrhage in case reports, interim analysis or post hoc analysis of randomized controlled trials.[1, 2, 5–12, 20]

Methylthiadiazolethiol (MTDT)-side-chain-containing cephalosporins such as cefazolin^21^ or n-hydroxyethyltetrazolethiol (HTT)-side-chain-containing cephalosporins such as flomoxef were found to result in inhibition of vitamin K-epoxide reductase in rats. In addition, cefoxitin, which is without any heterocyclic side chains attached to 3-position of cephem nucleus, has been reported to cause hypoprothrombinemia.[3]

However, most of the previous studies are limited by the small sample size and assessment of a single cephalosporin. In particular, it is uncertain that whether hypoprothrombinemia-related bleeding has a dose–response relationship. The aim of this nested case-control study was therefore to investigate the association between exposure to hypoprothrombinemia-inducing cephalosporins and the risk of hemorrhagic events with the use of the National Health Insurance Research Database (NHIRD) in Taiwan.

## Methods

### Data source

The National Health Insurance (NHI) program, with approximately 23 million insured persons, covers over 99% of Taiwan’s population. The NHIRD is a nationwide research database which contains comprehensive records of demographics and claims data that record healthcare utilization, including outpatient visits, hospital admissions, prescription medications, and diagnoses, of beneficiaries of Taiwan’s NHI program. The data we used in this study were the subset of the NHIRD, the Longitudinal Health Insurance Database (LHID), which contained all inpatient and outpatient medical claims for approximately three million individuals (approximately 15% of all beneficiaries) randomly selected from the Registry for Beneficiaries of the NHIRD. The distribution of age, gender, and average insured payroll-related amount do not differ between the LHID and the NHIRD.[21]

### Ethical statement

The protocol of our study was approved by the Research Ethics Committee of National Taiwan University Hospital (registration number, 201312061RINB). Because the identification numbers of all subjects in the NHRID were de-identified to protect individual privacy, informed consents were waived.

### Study cohort

We identified a cohort composed of patients aged 20 years or greater who used selected intravenous antibiotics for more than 48 hours in the emergency room (ER) between 2000 and 2011. We excluded those who switched antibiotic regimen during ER stay. Patients who had hemorrhage-related diagnoses or who used vitamin K1, fresh frozen plasma, and coagulation factors II, VII, IX, and X in the ER were excluded to avoid carry-over effect of hemorrhagic events. The cohort entry date for each patient was defined as the date when a selected intravenous antibiotic was first prescribed in the ER.

### Cases and Controls

From the study cohort, we identified patients hospitalized due to a hemorrhagic event subsequent to the use of antibiotics in the ER. A hemorrhagic event was defined as patients who hospitalized with a principal diagnosis codes of hemorrhage (*International Classification of Diseases*, *9th Revision*, *Clinical Modification codes (ICD-9-CM codes)*) or who used vitamin K1, fresh frozen plasma, or coagulation factors II, VII, IX, and X during hospitalization. A detailed list of the ICD-9-CM codes for hemorrhage ^24^ is presented in Appendix 1. The date of hospital admission was defined as the index date. We randomly selected 4 controls for each case from the study cohort according to an incidence-density sampling strategy. Controls were matched to cases for age (+/- 5 years), sex, and the cohort entry date (+/- 1 year).

### Exposure to Antibiotics

For each antibiotic a patient received at the ER visit, we further categorized them as hypoprothrombinemia-inducing cephalosporins and reference antibiotics. Hypoprothrombinemia-inducing cephalosporins include cefmetazole, cefoxitin, cefotetan, flomoxef, moxalactam, cefamandole, and cefoperazone, which have been reported to cause hypoprothrombinemia or hemorrhage in previous studies. [1, 2, 5–12, 20]. Antibiotics with similar clinical indications but no previous reports of hypoprothrombinemia or hemorrhage, including amoxicillin-clavulanic acid, ampicillin-sulbactam, cefuroxime, ceftriaxone, and cefotaxime, were defined as reference antibiotics. Cumulative dose of hypoprothrombinemia-inducing cephalosporins and reference antibiotics was calculated based on defined daily dose (DDD) [22], and divided into three categories as less than 3 DDD, 3–5 DDDs, and more than 5 DDDs.

Indications for antibiotic use in the ER included septicemia [ICD-9-CM code: 038, 0419, and 7907], lower respiratory tract infections [480–483, 485, 486, 487, 510, and 513], intra-abdominal infections [540–542, 56201, 56203, 56211, 56213, 566–567, 5695, 5720, 5721, and 5750], genitourinary tract infections [590, 5990, 601, and 614–616], skin and soft tissue infections [680–686], bone and joint infections [7110, and 730], and post-operation wound infections [9966, 9985, and 9993].[23] Using the outpatient and inpatient claims of the LHID, we identified the following comorbidities based on data within 6 months prior to the index date: overall comorbidity index calculated by Pharmacy-Based Disease Indicator (PBDI), chronic viral hepatitis, liver failure, renal disease, and malignancy. The PBDI, which was developed based on Chronic Disease Score (CDS), was an index consisting of 37 drug categories defined by Anatomical Therapeutic Chemical codes (ATC codes). It has also been validated in the NHIRD.[24]

Concomitant medications potentially associated with an altered hemorrhagic risk, including antiplatelet agents (ATC codes: B01AC and C04AD03), anticoagulants (B01AA-AB, B01AD-AE, and B01AX),[20] non-steroidal anti-inflammatory drugs (NSAIDs, M01AA-AC, M01AE, M01AG-AH, M01AX), drugs for chronic viral hepatitis (J05AB04, J05AF05, J05AF08, J05AF10-12, L03AB04-05, and L03AB09-11), drugs for liver failure (A06AD11, H01BA01, H01BA04, H01BA06, and H01CB01-02),[20, 25] drugs for renal disease (V03AE—and A11CC—), [3] and chemotherapeutic drugs (L01AA-AD, L01AG, L01AX, L01BA-BC, L01CA-CD, L01CX, L01DA-DC, L01XA-XE, L01XX-XY, L03AA, L03AC, and A04AA) [25] were also extracted from the NHIRD.

### Statistical analysis

Multivariate conditional logistic regression models were used to estimate the association between the use of hypoprothrombinemia-inducing cephalosporins and risk of hemorrhagic events. All models were adjusted for indications for antibiotic use in the ER and potential risk factors of hemorrhagic events (comorbidities and concomitant medications). We also adjusted for other risk factors of hemorrhagic events, including surgery or other invasive procedures, coagulopathy or hemorrhagic events, and poor nutritional status (defined as use of total parenteral nutrition (TPN), megestrol, or tube feeding[3]) within 6 months prior to the index date. Coagulopathy were defined as a group of cytopenic and coagulopathic-related diseases identified by ICD-9-CM codes, including secondary to blood loss (ICD-9-CM code: 280.0), acute posthemorrhagic anemia (285.1), coagulation defects (286.xx), purpura and other hemorrhagic conditions (287.xx), unspecified diseases of blood and blood-forming organs (289.9) and abnormal coagulation profile (790.92). To minimize selection bias regarding indications for antibiotics between the cases and controls, the model was adjusted by the propensity score of indications for antibiotics. The propensity score was assigned based on the probability that an individual would receive a study antibiotic or not and estimated by a multivariable logistic regression model adjusting for underlying indications of antibiotics (i.e. septicemia, lower respiratory tract infections, intra-abdominal infections, genitourinary tract infections, skin and soft tissue infections, bone and joint infections, or post-operation wound infections) of a patient.

The fundamental model-fitting techniques for stepwise variable selection with entry criterion of 0.1 and staying criterion of 0.15 were conducted in our regression analyses, which means only variables having a significant univariate test (crude OR) at these specified levels are selected as candidates for the multivariate analysis.

We also conducted regression diagnostics of multicollinearity. Associations are presented as adjusted odds ratios (aORs) with confidence intervals stated at the 95% confidence level. All data in this study were analyzed using SAS^®^ software, version 9·2 (SAS Institute, Cary, NC, USA).

## Results

**Fig 1:**
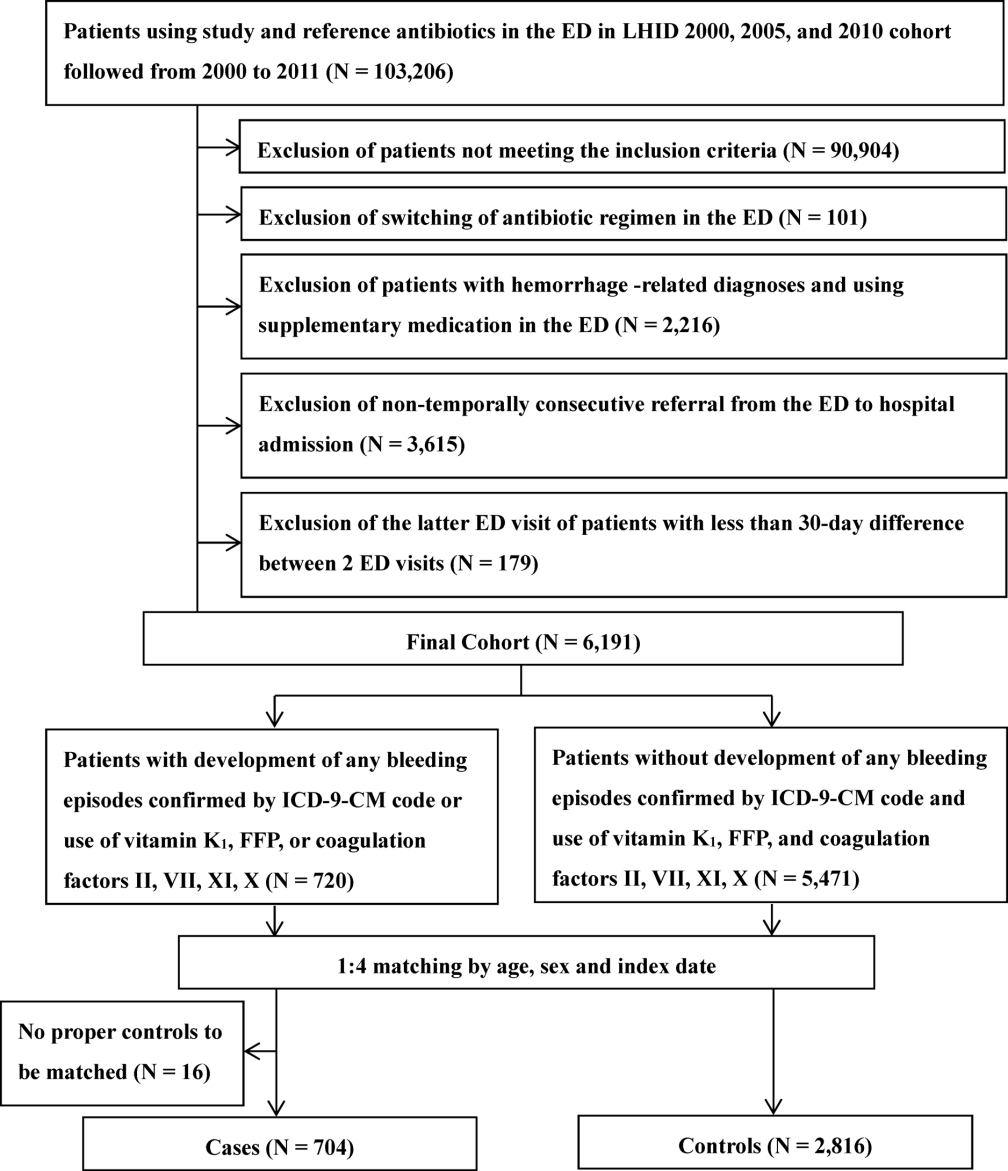
Flow chart describing the enrollment of the study cohort, the cases and the controls.

Among 6191 patients in the cohort, 704 case patients with hemorrhagic events and 2816 matched controls were identified (**Fig 1**). The mean age was 68 years and more than 60% (64.9%) were male (64.9%). In general, the case patients and controls shared a similar pattern with respect to indications for antibiotic use in the ER. However, case patients had significantly more frequent comorbidities than the controls, which was reflected by the PBDI (1.79 ± 1.29 versus 1.60 ± 1.29). Case patients were more likely to be diagnosed with coagulopathy (2.41% versus 0.46%) and hemorrhage (15.77% versus 5.82%), to undergo surgery or invasive procedures (48.44% versus 35.83%), and to have poor nutritional status (65.20% versus 51.31%) within 6 months prior to the index date. More case patients than controls used anticoagulants (20.60% versus 10.80%), NSAIDs (57.95% versus 54.72%), antiviral therapy for chronic viral hepatitis (1.85% versus 0.64%), medications for liver failure (17.47% versus 8.95%) and renal disease (7.39% versus 5.72%), and chemotherapy (10.23% versus 7.71%) within 6 months prior to the index date (**Table 1**).

**Table 1:** Characteristics of cases and controls in the cohort.

Characteristics	Cases	Controls	*p*-value
	(N = 704)	(N = 2816)	
	n (%)	n (%)	
Gender			
Male	454 (64.49)	1816 (64.49)	**--**
Female	250 (35.51)	1000 (35.51)	**--**
Age, mean [SD]	67.64 [16·04]	67.72 [16·60]	**--**
20–39 years	44 (6.25)	195 (6.92)	**--**
40–64 years	218 (30.97)	872 (30.97)	**--**
65 years and older	442 (62.78)	1749 (62.11)	**--**
Indications for antibiotics			
Septicemia	77 (10.94)	307 (10.90)	1.000
Lower respiratory tract infections	155 (22.02)	807 (28.66)	<0.0001
Intra-abdominal infections	34 (4.83)	138 (4.90)	0.951
Genitourinary tract infections	75 (10.65)	333 (11.83)	0.163
Skin and soft tissue infections	24 (3.41)	195 (6.92)	<0.0001
Bone and joint infections	2 (0.28)	7 (0.25)	1.000
Post-operation wound infections	0 (0.00)	3 (0.11)	1.000
Prior medical conditions*			
PBDI	1.79 (1.29)	1.60 (1.29)	<0.0001
Coagulopathy	17 (2.41)	13 (0.46)	<0.0001
Hemorrhagic events	111 (15.77)	164 (5.82)	<0.0001
Cerebrovascular hemorrhage	7 (0.99)	15 (0.53)	0.066
Gastrointestinal hemorrhage	67 (9.52)	90 (3.20)	<0.0001
Other types of hemorrhage	52 (7.39)	80 (2.84)	<0.0001
Surgery or invasive procedure	341 (48.44)	1009 (35.83)	<0.0001
Poor nutritional status	459 (65.20)	1445 (51.31)	<0.0001
Prior medications*			
Anticoagulants	145 (20.60)	304 (10.80)	<0.0001
Antiplatelet agents	182 (25.85)	757 (26.88)	0.374
NSAID	408 (57.95)	1541 (54.72)	0.015
Drugs for chronic viral hepatitis	13 (1.85)	18 (0.64)	<0.0001
Drugs for liver failure	123 (17.47)	252 (8.95)	<0.0001
Drugs for renal disease	52 (7.39)	161 (5.72)	0.014
Chemotherapeutic drugs	72 (10.23)	217 (7.71)	<0.0001

*Prior medications and prior medical conditions were documented within six months prior to the index date.

Abbreviations: NSAIDs, nonsteroidal anti-inflammatory drugs; PBDI, Pharmacy-Based Disease Indicator; SD, standard deviation

More case patients used hypoprothrombinemia-inducing cephalosporins (38.49% versus 27.49%). Among all hypoprothrombinemia-inducing cephalosporins, flomoxef was the most frequently used (24.01% and 20.81% in case patients and controls, respectively). No patients in our study used cefotetan, moxalactam, and cefamandole in the ER. In contrast, more controls than case patients (72.51% versus 61.51%) used reference antibiotics. Among all reference antibiotics, ampicillin/sulbactam (27.27% and 30.54% in case patients and controls, respectively) was the most frequently used, followed by cefuroxime (12.22% and16.23% in case patients and controls, respectively) (**Table 2**).

**Table 2:** The comparison between the cases and the controls regarding use of study and reference antibiotics.

Characteristics	Cases	Controls	*p*-value
	(N = 704)	(N = 2816)	
	n (%)	n (%)	
Hypoprothrombinemia-inducing cephalosporins	271 (38.49)	774 (27.49)	<0.0001
Cefmetazole	67 (9.52)	130 (4.62)	<0.0001
Cefoxitin	6 (0.85)	31 (1.10)	0.410
Flomoxef	169 (24.01)	586 (20.81)	0.004
Cefoperazone	29 (4.12)	27 (0.96)	<0.0001
Reference antibiotics	433 (61.51)	2042 (72.51)	<0.0001
Amoxicillin/clavulanate	111 (15.77)	608 (21.59)	<0.0001
Ampicillin/sulbactam	192 (27.27)	860 (30.54)	0.007
Cefuroxime	86 (12.22)	457 (16.23)	<0.0001
Cefotaxime	18 (2.56)	48 (1.70)	0.032
Ceftriaxone	26 (3.69)	69 (2.45)	0.008

The conditional logistic regression model showed that the increased risk of hemorrhagic events was associated with exposure to hypoprothrombinemia-inducing cephalosporins (aOR 1.71; 95% CI, 1.42–2.06). In addition, higher hemorrhagic risks were found in patients who used anticoagulants (aOR 2.08; 95% CI, 1.64–2.63), had liver failure (aOR 1.69; 95% CI, 1.30–2.18), were in poor nutritional status (aOR 1.41 95% CI, 1.15–1.73), and had a history of hemorrhagic events (aOR 2.57; 95% CI, 1.94–3.41) (**Table 3**).

**Table 3:** Risk factors of hemorrhagic events associated with use of study antibiotics.

		Hemorrhagic events		
	Crude OR	*p*-value	Adjusted OR*	*p*-value
	(95% CI)		(95% CI)	
Use of hypoprothrombinemia-inducing cephalosporins	1.70 (1.42–2.03)	<0.0001	1.71 (1.42–2.06)	<0.0001
Prior medication^+^				
Anticoagulants	2.16 (1.73–2.69)	<0.0001	2·08 (1.64–2·63)	<0.0001
Drugs for liver failure	2.13 (1.69–2.69)	<0.0001	1.69 (1.30–2.18)	<0.0001
Drugs for chronic viral hepatitis	3.04 (1.46–6.36)	0.003	2.01 (0.91–4.42)	0.084
Antiplatelet agents	0.94 (0.76–1.15)	0.563		
NSAIDs	1.15 (0.97–1.36)	0.116		
Drugs for renal disease	1.31 (0.95–1.81)	0.100		
Chemotherapeutic drugs	1.39 (1.04–1.85)	0.026		
Prior medical events^+^				
Hemorrhagic events	3.01 (2.38–4·03)	<0.0001	2.57 (1.94–3.41)	<0.0001
Poor nutritional status	1.79 (1.50–2.12)	<0.0001	1.41 (1.15–1.73)	0.001
Severity of illness calculated by PBDI	1.12 (1.05–1.20)	0.0006	0.92 (0.85–0.99)	0.038
Surgery or invasive procedure	1.69 (1.43–2.00)	<0.0001		
Coagulopathy	5.23 (2.54–10.77)	<0.0001		
Age	0.99 (0.96–1.03)	0.723		
Indications for antibiotics calculated by propensity score^#^	2.04 (1.30–3.20)	0.002		

*adjusted by use of anticoagulants, drugs for liver failure, and drugs for chronic viral hepatitis, occurrence of hemorrhagic events, and poor nutritional status within 6 months prior to the index date, and severity of illness calculated by PBDI within 6 months prior to the index date

Abbreviations: NSAIDs, nonsteroidal anti-inflammatory drugs; OR, odds ratio; CI, confidence interval; PBDI, Pharmacy-Based Disease Indicator

Prior medication and prior medical events^+^: within 6 months prior to the index date

# The propensity score was assigned based on the probability that an individual would receive a study antibiotic or not and estimated by a multivariable logistic regression model adjusting for underlying indications of antibiotics (i.e. septicemia, lower respiratory tract infections, intra-abdominal infections, genitourinary tract infections, skin and soft tissue infections, bone and joint infections, or post-operation wound infections) of a patient.

The association with hemorrhagic risks was stronger with higher cumulative exposure to hypoprothrombinemia-inducing cephalosporins, for which the aOR increased from 1.62 (95% CI, 1.28–2.05) for those with <3 DDDs, to 1.78 (95% CI, 1.35–2.34) for 3–5 DDDs, and to 1.89 (95% CI, 1.28–2.78) for those with >5 DDDs of hypoprothrombinemia-inducing cephalosporins (**Table 4**).

**Table 4:** Dose-dependent effect regarding association between use of study antibiotics and hemorrhagic tendency/events.

	N (%)	Crude OR (95% CI)	*p*-value	Adjusted OR*(95% CI)	*p*-value
Reference antibiotics	2475	Reference		Reference	
Cumulative dose of study antibiotics					
< 3 DDD	558	1.58 (1.26–1.97)	< 0·0001	1.62 (1.28–2.05)	< 0·0001
≥ 3 DDD, < 5 DDD	336	1.80 (1.38–2.35)	< 0·0001	1.78 (1.35–2.34)	< 0·0001
≥ 5 DDD	151	1.94 (1.34–2.82)	0·0005	1.89 (1.28–2.78)	0·0013

*adjusted by use of anticoagulants, drugs for liver failure, and drugs for chronic viral hepatitis, occurrence of hemorrhagic events, and poor nutritional status within 6 months prior to the index date, and severity of illness calculated by PBDI

Abbreviations: DDD, defined daily dose; ER, emergency room; OR, odds ratio; CI = confidence interval

For individual hypoprothrombinemia-inducing cephalosporin, cefoperazone (aOR 4.57; 95% CI, 2.63–7.95), cefmetazole (aOR 2.88; 95% CI, 2.08–4.00) and flomoxef (aOR 1.35; 95% CI, 1.09–1.67) were all associated with increased risk of hemorrhagic event. No such association was found in users of cefoxitin (aOR 0.83; 95% CI, 0.33–2.08) (**Table 5**).

**Table 5:** Risk of hypoprothrombinemia or hemorrhagic events associated with use of individual study antibiotics.

	Cases	Controls	Crude OR (95% CI)	*p*-value	Adjusted OR*(95% CI)	*p*-value
	(N = 704)	(N = 2816)				
Reference antibiotics	433	2042	Reference		Reference	
Cefmetazole	67	130	2.46 (1.80–3.36)	<0·0001	2.88 (2.08–4.00)	<0·0001
Flomoxef	169	586	1.39 (1.13–1.71)	0·002	1·35 (1.09–1·67)	0.006
Cefoxitin	6	31	0.85 (0.34–2.11)	0.73	0.83 (0.33–2.08)	0.69
Cefoperazone	29	27	4.93 (2.89–8·41)	<0·0001	4.57 (2.63–7.95)	<0·0001

*adjusted by use of anticoagulants, drugs for liver failure, and drugs for chronic viral hepatitis, occurrence of hemorrhagic events, and poor nutritional status within 6 months prior to the index date, and severity of illness calculated by PBDI

## Discussion

In this population-based nested case-control study, we found an increased risk of hemorrhagic events associated with hypoprothrombinemia-inducing cephalosporins, which was demonstrated in a dose-response relationship with the highest risks seen in hypoprothrombinemia-inducing cephalosporins users with >5 DDDs in the ER. We also found that cefoperazone and cefmetazole (NMTT-side-chain-containing cephalosporins) were associated with higher risks of hemorrhagic events. This phenomenon was consistent with the half-life of prothrombin (clotting factor II) and its major effect in the coagulation cascade.[17,18] The risk of hemorrhagic events was statistically significantly increased in patients who used anticoagulants as well as in patients with liver failure, poor nutritional status, and history of hemorrhagic events.

Our findings on the dose-response relationship of hypoprothrombinemia-inducing cephalosporins with increased hemorrhagic risks are in line with the only study so far that assessed the associations between hypoprothrombinemia and bleeding events with exposure to a higher cumulative dose of cefoperazone.[20] Furthermore, with the use of the Taiwanese NHIRD, we are able to provide more comprehensive analysis of the risk of hemorrhagic events among an Asian population who receive hypoprothrombinemia-inducing cephalosporins for a variety of clinical indications.

Previous studies that investigated the hemorrhagic events among patients receiving hypoprothrombinemia-inducing cephalosporins are limited by small sample sizes. [1,5,20] By using the nationwide data, we had access to a larger cohort who used hypoprothrombinemia-inducing cephalosporins than existing studies, which allowed us to compare the risk of hemorrhagic events among different hypoprothrombinemia-inducing cephalosporins. The findings that cefoperazone, cefmetazole and flomoxef were at the highest risk among all antibiotics studied, and that the risks of cefoperazone and cefmetazole were much higher than that of flomoxef can be inferred from the previous study indicating that inhibition of vitamin K epoxide reductase caused by NMTT side chain was more intense than that by HTT side chain.[19]

In this study, we found that cefoperazone and cefmetazole (NMTT-side-chain-containing cephalosporins) were associated with 4.5 folds and 2.8 folds higher risk of bleeding events, respectively. Cefoperazone, the most commonly reported to cause hypoprothrombinemia in the previous studies, has been found to result in PT prolongation for more than 10 seconds in 2 case reports.[10, 11] In addition, 2 retrospective cohort studies also suggested that patients receiving cefoperazone were more likely to develop PT prolongation or hemorrhage than those receiving ceftizoxime/cefotaxime and ceftazidime.[4, 20]

In contrast, we did not found an increased hemorrhagic risk associated with another hypoprothrombinemia-inducing cephalosporin, cefoxitin. This is inconsistent with a retrospective cohort study done by Brown et al, in which they reported that cefoxitin users were more likely to experience hemorrhagic events than users of cefazolin, chloramphenicol, erythromycin, nafcillin or oxacillin, and vancomycin.[3] Small case numbers of patients with exposure to cefoxitin in our study could be a potential explanation as only 6 case patients and 31 controls received cefoxitin.

Our study identified several important risk factors of hemorrhagic event among our study subjects. In addition to common risk factors of bleeding events such as use of anticoagulants or history of hemorrhagic event, we found that poor nutritional status was associated with a 40% increase of risk of hemorrhagic event (aOR 1.41; 95% CI, 1.15–1.73). These findings gave support to a case report that demonstrated a patient with poor nutritional status and end-stage renal disease who was noted to have elevated international normalized ratio (INR) level after receiving post-surgery cefmetazole.[2]

Some limitations of this study should be acknowledged. First, the patients who were hospitalized without receiving care at the ER were not included in this analysis. Second, we were unable to include variables not routinely captured in claims database, such as laboratory data. We thus were unable to identify INR prolongation as our study endpoint or identify hypoalbuminemia as one of the covariates. Instead, we retrieved use of vitamin K, FFP, or coagulation factors II, VII, IX, and X as surrogates for INR prolongation, and use of TPN, megestrol, and tube feeding for poor nutritional status. We also may not fully capture platelet dysfunction for our study cohort. However, we have retrieved our data and found none of our cases with bleeding and controls had a diagnosis of thrombocytopenia nor do they ever received platelet transfusion. In addition, covariates which can affect a subject’s platelet number or function were considered in our statistical models. We believe we have done our best to control the potential differences in covariates between our cases and control.

Third, dose adjustment of antibiotics due to renal impairment or antibiotic regimens for diverse infectious diseases with severity that could vary widely may not be well captured in the NHIRD. However, we used DDDs to calculate cumulative doses of antibiotics, which is commonly used for measurement of exposure to different medications in studies using claims data. Fourth, we did not consider cefazolin in our study, which was mainly due to the different antimicrobial spectrums between cefazolin and our study antibiotics (cefmetazole, cefoxitin, cefotetan, flomoxef, moxalactam, cefamandole, and cefoperazone). Including cefazolin as one of our study antibiotics may result in diverse discrepancies, in terms of indications and clinical characteristics, among our study subjects. In addition, existing evidence regarding cefazolin-associated hypoprothrombinemia is very limited as this potential association was only reported in one case report [4]. Nevertheless, further studies regarding cefazolin-associated hypoprothrombinemia are warranted.

In conclusion, we found an increased risk of hemorrhagic events associated with exposure to hypoprothrombinemia-inducing cephalosporins, especially NMTT-side-chain-containing cephalosporins (cefoperazone and cefmetazole). Close monitoring of INR levels is recommended mainly in patients who are on anticoagulants, in poor nutritional status, or with underlying liver failure or a history of hemorrhagic events.
